# Does healthcare voucher provision improve utilisation in the continuum of maternal care for poor pregnant women? Experience from Bangladesh

**DOI:** 10.1080/16549716.2019.1701324

**Published:** 2019-12-11

**Authors:** Shehrin Shaila Mahmood, Mark Amos, Shahidul Hoque, Mohammad Nahid Mia, Asiful Haidar Chowdhury, Syed Manzoor Ahmed Hanifi, Mohammad Iqbal, William Stones, Saseendran Pallikadavath, Abbas Bhuiya

**Affiliations:** a Health Systems and Population Studies Division, icddr,b, Dhaka, Bangladesh; b Portsmouth Brawajaya Centre for Global Health, University of Portsmouth, Portsmouth, UK; c IMPACT study, ARK Foundation, Dhaka, Bangladesh; d College of Medicine, University of Malawi, Blantyre, Malawi

**Keywords:** Maternal health, continuum of care, poor pregnant women, antenatal care, postnatal care, cluster analysis, maternal health voucher scheme

## Abstract

**Background**: Improving maternal health is a major development goal, with ambitious targets set for high-mortality countries like Bangladesh. Following a steep decline in the maternal mortality ratio over the past decade in Bangladesh, progress has plateaued at 196/100,000 live births. A voucher scheme was initiated in 2007 to reduce financial, geographical and institutional barriers to access for the poorest.

**Objective**: The current paper reports the effect of vouchers on the use of continuum of maternal care.

**Methods**: Cross-sectional surveys were carried out in the Chattogram and Sylhet divisions of Bangladesh in 2017 among 2400 women with children aged 0–23 months. Using Cluster analysis utilisation groups for antenatal care, facility delivery and postnatal care were formed. Clusters were regressed on voucher receipt to identify the underlying relationship between voucher receipt and utilisation of care while controlling for possible confounders.

**Results**: Four clusters with varying levels of utilisation were identified. A significantly higher proportion of voucher-recipients belonged to the high-utilisation cluster compared to non-voucher recipients (43.5% vs. 15.4%). For the poor voucher recipients, the probability of belonging to the high-utilisation cluster was higher compared to poor non-voucher recipients (33.3% vs. 6.8%) and the probability of being in the low-utilisation cluster was lower than poor non-voucher recipients (13.3% vs. 55.4%).

**Conclusion**: The voucher programme enhanced uptake of the complete continuum of maternal care and the benefits extended to the most vulnerable women. However, a lack of continued transition through the continuum of maternal care was identified. This insight can assist in designing effective interventions to prevent intermittent or interrupted care-seeking. Programmes that improve access to quality healthcare in pregnancy, childbirth and the postnatal period can have wide-ranging benefits. A coherent continuum-based approach to understanding maternal care-seeking behaviour is thus expected to have a greater impact on maternal, newborn and child health outcomes.

## Background

Improving maternal health is one of the major global goals, particularly for low-resource countries like Bangladesh. The current Sustainable Development Goals articulate an aim to reduce maternal mortality to less than 70 per 100,000 live births by the year 2030 [1]. In Bangladesh, although there has been a steep decline in the maternal mortality ratio during the past decade, it has plateaued at around 196 per 100,000 live births during 2008–2016 [2]. Barriers such as out-of-pocket expenses, inequitable access to services and delay in care-seeking have been associated with the risk of maternal complications [3]. Evidence from the 2014 Bangladesh Demographic and Health Survey shows that despite the increase in rates of facility delivery, home birth is still the norm. Given that the health system in rural Bangladesh is not configured to provide skilled delivery care at home, there is a continued reliance on informal healthcare providers, such as traditional birth attendants. Further, inadequate and poor-quality maternal healthcare, including antenatal care (ANC), delivery, and postnatal care (PNC), can lead to poor maternal health outcomes [4]. ANC, skilled birth attendants (SBA) and PNC have been shown to reduce the risk of maternal deaths [5]. PNC, especially within the first 48 hours of delivery is critical in detecting and managing maternal and newborn complications such as sepsis and difficulties with establishing lactation. Each of these interventions is important and should be included in a continuum of maternal care (CoC) to ensure the health of both the mother and the newborn. Development partners including the World Health Organization have been advocating a focus on the CoC over the past decade to improve maternal, newborn and child health [6] and it has become a key element in programme implementation strategies.

A range of interventions has been tested to overcome barriers to access for maternal health services among marginalised groups such as the poor. These interventions have targeted both demand and supply-side issues. Voucher schemes, a demand-side financing instrument, have been introduced in the health sector of a number of low- and middle-income countries to increase demand for services among targeted groups by reducing healthcare expenses for households at risk of not seeking care in the absence of the subsidy [7]. This approach includes interventions that channel government or donor subsidies predominantly to service users rather than to service providers [7]. The aim is to incentivise certain positive behaviours such as in-facility childbirth or medical check-ups during pregnancy, either through direct cash payments, or by subsidising service providers to allow free access.

Bangladesh introduced a voucher scheme in 2007, popularly known as the Maternal Health Voucher Scheme (MHVS), as a programmatic response to reducing financial, geographical and institutional barriers to accessing maternal health services. The scheme targets poor pregnant women who are identified based on household income and land ownership criterion. The benefits of the scheme span the complete CoC including ANCs, delivery care and PNCs [8].

A few evaluation studies of the MHVS explored the impact of the scheme on the individual components of the CoC in isolation but not on the continuum as a whole [7,9–12]. Further, all the studies were based on evaluation in the initial years of implementation and were not able to capture longer-term impacts [13]. To date, global literature on voucher schemes has mostly focused on either one component of the CoC [14,15] or has examined the flow of women from one stage of the continuum to another, for example, from ANC to delivery [14,16–18]. A limitation is that this does not allow the evaluation of voucher provision on the receipt of all CoC components, nor does it allow the identification of dropouts from the scheme [19]. Also, the potential underlying correlations between the utilisation of different components of CoC cannot be examined [20]. Successful programme implementation to improve the CoC relies on a better understanding of where the gaps are in the care-seeking pathway and what factors may contribute to those gaps. Therefore, the effect of vouchers (i.e. MHVS) on ensuring the entirety of the CoC for voucher recipients was analysed. A novel methodological approach has been used to examine care utilisation across the entire pregnancy and post-pregnancy timeframe and identify distinct subgroups for care utilisation. Although the term “continuum of care” originally refers to the continuity of care throughout the lifecycle – adolescence, pregnancy, childbirth, post-delivery period, and childhood [6], the current study focuses just on the continuum of care through pregnancy, childbirth and postnatal period. The aim was to link predictor variables, such as voucher receipt, to the utilisation of the CoC to assess the effect of the scheme across the pregnancy, delivery and postnatal period.

## Methods

*The Maternal Health Voucher Scheme (MHVS)* of the Government of Bangladesh was initiated in 21 sub-districts in 2007, and currently operates in 53 of the country’s 556 sub-districts. A targeted voucher scheme meant for poor pregnant women (having a maximum household monthly income of Bangladeshi Taka 2500 (approximately US Dollar 30/month)) the MHVS covers three ANCs, delivery at a health facility, one PNC, management of maternal complications including caesarean delivery where required, free medicines, cash allowances for transportation, and a cash incentive to deliver at a health facility. Provider facilities and staff also receive a payment for each service provided to the scheme participants [10,21]. The voucher can be used in both public hospitals and designated private facilities. Details of the benefit package are provided in Table 1.

**Table 1.** Benefit package of MHVS.

Description of Services	Reimbursement to provider (BDT)	Incentive to beneficiaries
Registration of an eligible woman	20	
ANC: Two blood and 2 urine tests	(35x4) = 140	Free service
Three ANC check-ups (at facility)	(50x3) = 150	Free service+ transportation (BDT 300 for 3 visits)
One PNC check-up (at facility)	50	Free service+ transportation (BDT 100)
Normal delivery	300	BDT 2000 (at facility)/BDT 500 (home with CSBA) + transportation (BDT 100)
Medicines	100	Free medicine
Forceps delivery, manual removal of placenta, dilation and curettage, or vacuum extraction	1000	Free service + emergency transportation to referral facility, if needed (ceiling BDT 500)
Medicines for management of eclampsia	1000	
C-section and medicine	6000	BDT 2000 (qualified provider).

Except for medicines and registration incentives, 50% of provider reimbursement goes to a ‘seed fund’ used to improve the quality of care at the facility. The remaining 50% is distributed among people directly involved in providing services. This does not apply to private facilities where providers receive full reimbursement.

### Study design and study site

A cross-sectional study was carried out during October 2016 till June 2017 to observe the difference in service utilisation between voucher and non-voucher participants in terms of utilisation of the CoC. The study was conducted in two of the low-performing (in terms of maternal health outcome) divisions of Bangladesh – Chattogram and Sylhet. In Chattogram, two sub-districts, Ramu and Teknaf, were randomly selected from the total 11 voucher areas. In Sylhet, two sub-districts, Srimongal and Salla, were randomly chosen from the five voucher areas.

In both areas, public and private healthcare facilities are available to provide institutional delivery. In addition, SBAs as well as traditional birth attendants conduct home deliveries. The percentage of women delivering at home is very high in both areas (76.6% in Sylhet, 64.4% in Chattogram) compared to the national rate of 62.2% [3].

### Sample size and respondents

From each of the four sites in Chattogram and Sylhet, 600 women with children aged 0–23 months were selected. In the two voucher areas of Chattogram, 1446 eligible women were identified and 1200 were randomly selected for interview. In the two voucher areas of Sylhet, 1502 women were eligible and 1200 were randomly selected for interview. Finally, from all four sites, a total of 2400 women with children aged 0–23 months were interviewed.

### Data collection

A team of 20 female interviewers with 12 years of education collected data. A four-day training was provided by the statistician to the data collection team and the questionnaire was pre-tested before data collection. Two field supervisors and one statistician supervised the data collection process. Responses were recorded directly on tablets using Open Data Kit software. Features were built into the software to reduce the likelihood of errors in data entry. To detect any anomalies, the quality control team (consisting of one quality control officer and three re-interviewers) re-visited 5% of the households, chosen randomly, within 2 days of data collection. The supervisors, data collectors and the quality control team met at regular intervals to resolve inconsistencies in the data. All completed questionnaires were checked for completeness and inconsistencies. Subsequently, the statistician used computer-based data editing procedures to confirm the quality of the data.

### Data analysis

#### Cluster analysis

Cluster analysis was used to allow the formation of meaningful groupings of care utilisation across the CoC using indicators for ANC, delivery and PNC. Indicator variables were created for ANC. These were whether a woman had the recommended number of ANC visits (3 or more visits) and whether a woman had some ANC care but too few visits (1–2 visits). Delivery included whether the woman had an SBA present and whether the birth took place in a facility. For PNC the variable was whether the mother received a check within 2 days of delivery. All the variables were binary, taking the values ‘1ʹ if the woman received the care and ‘0ʹ if not. A measure of dissimilarity was then created where women with similar levels of care utilisation had smaller dissimilarity and women with different care utilisation had higher dissimilarity. The cluster analysis was performed using the cluster function in Stata 13.0 for Windows using Ward’s linkage.

*A person-centred approach* was followed to identify patterns of care for individual women within the dataset and to form meaningful care utilisation clusters that were linked to respondents’ characteristics. The person-centered approaches treat the individual as the unit of analysis, seeking to form groupings of persons with similar characteristics, rather than similar profiles of variables [22]. Once clusters within the data were identified, these were then used as response variables within a multinomial logistic regression to allow identification of the key factors predicting cluster membership. The clusters were regressed on receipt of the voucher to identify any underlying relationship between voucher receipt and care utilisation after controlling for distance to health facilities, parity, mother’s educational attainment and mother’s age. Predicted probabilities of cluster membership by voucher receipt status were generated to aid interpretation.

### Definition of variables

#### Socioeconomic status

In the absence of household income data, household ‘socioeconomic status’ was defined using an asset index. Asset information of all study participants was collected which was then used to calculate the asset index. Based on the asset index, each household was classified into quintiles where the first quintile consists of the poorest 20% of households and the fifth quintile consists of the wealthiest 20% of households [23]. For the sake of analysis, women who belonged to the bottom two asset quintiles were considered as poor, i.e. the target population for the voucher scheme.

A Skilled Birth Attendant (SBA) is an ‘accredited health professional – such as a midwife, doctor or nurse – who has been educated and trained to proficiency in the skills needed to manage normal (uncomplicated) pregnancies, childbirth and the immediate postnatal period, and in the identification, management and referral of complications in women and newborns’ [24]. In rural Bangladesh, SBAs conduct deliveries both at home and at health facilities.

## Results

Factors that significantly influenced the probability of belonging to a particular utilisation cluster included: voucher receipt, socioeconomic status, age and education of mothers, distance of household to the health facility and birth order. A higher proportion of voucher-recipient mothers belonged to the high-utilisation cluster (43.5%) compared to those without vouchers (15.4%), whereas a significantly lower proportion of voucher-recipient women belonged to the low-utilisation cluster compared to non-voucher recipients (9.8% vs. 41.9%). A comparatively higher proportion of mothers in the highest socioeconomic group belonged to the high-utilisation cluster (41.8%) and a comparatively higher proportion of mothers from the lowest socioeconomic group (53.7%) belonged to the low-utilisation cluster. In the high-utilisation cluster, the proportion of younger mothers was higher, and in the low-utilisation cluster, mothers aged 35 or more occupied the major share (45.9%). The proportion of mothers with higher educational attainment was higher in the high-utilisation cluster (80.0%) by contrast with mothers with lower educational attainment representing a higher proportion in the low-utilisation cluster (48.4%). Similarly, mothers residing closer to the health facility were proportionately over-represented in the high-utilisation cluster (20.5%) and mothers living far from health facilities were over-represented in the low-utilisation cluster (36.3%) (Table 2).

**Table 2.** ANC, delivery, and PNC use among the different utilisation clusters, 2017.

Variables	Category	Cluster 1 (High utilisation 22.9%) % (n)	Cluster 2 (High utilisation, except ANC 6.1%) % (n)	Cluster 3 (Sufficient ANC, else low 37.3%) % (n)	Cluster 4 (Overall low utilisation 33.3%) % (n)
Voucher receipt (n = 2400)***	Yes	43.5 (199)	5.0 (23)	41.7 (191)	9.8 (44)
	No	15.4 (299)	6.5 (127)	36.2 (703)	41.9 (814)
ANC (n = 2400)**	3 or more	37.9 (510)	0.00 (0)	62.1 (835)	0.00 (0)
	1–2	0.0 (0)	18.9 (113)	0.0 (0)	81.0 (484)
	None	0.0 (0)	9.6 (44)	1.1 (5)	89.1(408)
Skilled birth attendant(n = 2400)**	Yes	49.9(519)	13.3 (139)	27.1(282)	9.6 (99)
	No	0.0 (0)	0.0 (0)	46.6 (634)	53.4 (726)
Facility birth(n = 2400)**	Yes	74.1 (519)	19.8 (139)	4.9 (34)	1.1 (8)
	No	0.0 (0)	0.0 (0)	52.3 (889)	47.6 (810)
Had a postnatal check within 2 weeks (n = 2400)**	Yes	58.1 (531)	15.5 (142)	21.0 (192)	5.3 (49)
	No	0.0 (0)	0.0 (0)	48.5 (720)	51.5 (765)
Socioeconomic status (n = 2400)***	Highest quintile (better-off)	41.8 (221)	9.6(51)	35.9 (190)	12.5 (66)
	4^th^ Quintile	27.7 (96)	6.5 (23)	38.3 (132)	27.3 (94)
	3^rd^ quintile	16.7 (97)	5.8(34)	39.0(227)	38.3(233)
	2^nd^ quintile	17.8(85)	3.7(19)	41.0(159)	37.3(251)
	lowest quintile (poorest)	8.3(39)	4.1(19)	33.8(159)	53.6(251)
Age group of mothers (n = 2400)***	Under 20	22.1(50)	4.9(11)	40.3(90)	32.6 (73)
	20–24	27.8 (213)	6.3 (49)	38.0(292)	27.8 (213)
	25–29	22.4 (160)	5.4 (38)	37.6(268)	34.5(246)
	30–34	21.4(103)	6.7(32)	35.8(172)	35.8(172)
	35 and more	9.0 (20)	8.2 (18)	36.8 (80)	45.8 (99)
Education of mothers*** (n = 2400)	Illiterate	11.6 (75)	3.7 (24)	36.1 (233)	48.4 (312)
	Primary	167 (16.9)	7.1 (70)	39.5 (391)	36.4 (360)
	Secondary	36.6 (251)	6.9 (47)	37.8 (259)	18.5 (127)
	Higher secondary	62.1(52)	5.4 (5)	29.7 (25)	2.7 (2)
	Graduate	80.0 (19)	6.6 (1)	6.6 (1)	6.6 (1)
Distance to health facility (n = 2400)***	Municipality (city center)	20.4 (23)	8.4 (9)	26.5 (29)	44.5 (49)
	Non-municipality but within 5km of health facility	35.9 (157)	5.5 (24)	37.5 (164)	20.9 (91)
	Non-municipality and beyond 5km of health facility	19.1 (355)	6.1 (114)	38.4 (712)	36.2 (672)
Birth order of current birth (n = 2400)***	1	31.8 (232)	6.5 (48)	39.5 (288)	22.0 (161)
	2	25.0 (175)	5.9 (41)	35.2 (245)	33.9 (236)
	3	20.3 (93)	8.2 (37)	40.1 (184)	31.4 (143)
	4	12.4 (32)	4.8 (12)	38.2 (99)	44.6 (116)
	≥5	6.3 (16)	3.4 (9)	34.1 (86)	56.3 (143)

***significant at 5% level. **significant at 10% level.

### Levels of service utilisation

**Figure 1.**
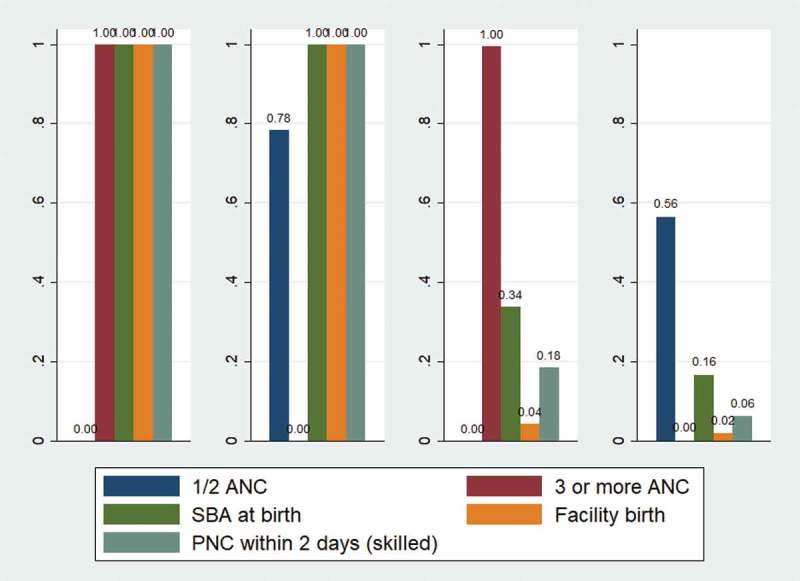
Cluster profile for 4 clusters solution by care utilisation pattern.

Figure 1 presents the different clusters of pregnant women based on their level of service utilisation where each of the bars within a cluster represent the proportion of women using a particular service (e.g. 1–2 ANCs, 3 or more ANCs, SBA at delivery, facility delivery and PNC within 2 days of delivery).

Women with high utilisation of all services were categorised as ‘High utilisation’ cluster (Cluster 1). Women in this cluster exhibit universal and complete service utilisation, with 100% of women having at least 3 ANC visits, giving birth in a facility assisted by an SBA and having PNC within 2 days of delivery. This cluster accounts for 22.9% of the sample.

Cluster 2 is titled ‘High utilisation, except ANC’. The major difference between this cluster and cluster 1 is that while a large proportion of women within this cluster make use of ANC visits (78.4%), they will only have a maximum of 2 ANC visits. The remaining women in this cluster receive no ANC at all, since they have no score recorded for either 3 or more, nor 1–2 ANC visits. These women account for 12% of the cluster. For the remainder of the continuum covering delivery and PNC services, care utilisation is high however, with 100% having an SBA present, 100% giving birth in a facility and 100% receiving PNC checks within 2 days of delivery. This cluster comprises 6.1% of women in the sample.

Cluster 3 is characterised by high care utilisation in the initial stage of the CoC (ANC visits), followed by some drop off and is hence titled ‘Sufficient ANC, else low’. 99.5% of women within this cluster received 3 or more ANC visits. However, only 33.8% of women within this cluster reported having an SBA present and only 4.3% reported giving birth in a facility (implying that even with SBA attendance the vast majority of births take place in the home). 18.4% of women within this cluster received PNC within 2 days of delivery. This cluster accounts for 37.3% of the total number of women in the sample.

The remaining cluster (cluster 4) constitutes women with overall low levels of care utilisation, and hence is titled ‘Overall low utilisation’. Whilst 56.5% of women in the cluster received some form of ANC, this amounts to a maximum of 2 ANC visits, with no woman receiving the recommended number of ANC checks (43.5% of women receive no ANC care whatsoever). Care utilisation for other stages of the CoC tails off rapidly, with 16.4% of women having an SBA at birth, 1.8% of women giving birth within a facility and 6% of women reporting a PNC within 2 days of delivery. This cluster contains 33.3% of the sample.

### Effect of voucher receipt on the probability of belonging to certain utilisation groups

**Figure 2.**
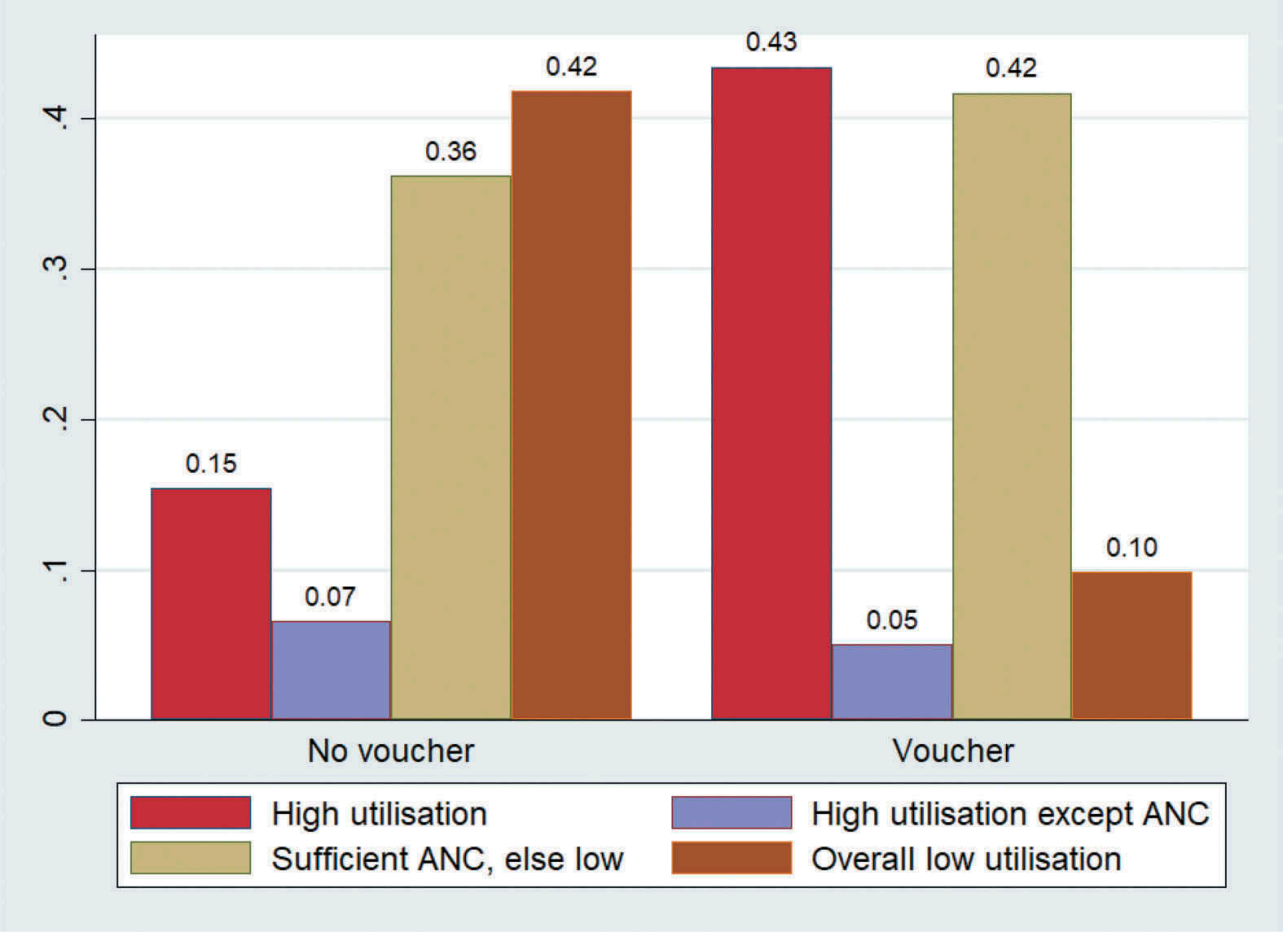
Predicted probabilities of cluster membership within all women.

Regression analysis was carried out to look into the effect of vouchers on membership in certain utilisation clusters. The results show a significant effect of voucher receipt on cluster membership, which was robust to the introduction of control variables. Predicted probabilities of cluster membership by voucher receipt status was plotted after controlling for other variables (Figure 2).

For non-voucher recipients, the predicted probability of being within cluster 1 (high utilisation) was 15.4%. The probability of membership of cluster 2 (high utilisation, except ANC) was lower (6.5%), while the probability of belonging to cluster 3 (sufficient ANC, else low) was higher at 36.1 and a similarly high predicted probability (41.8%) of belonging to cluster 4 (overall low utilisation) (Figure 2).

The predicted probabilities of cluster membership for voucher recipients showed that nearly half (43.4%) of voucher recipients were predicted to be members of cluster 1 (high utilisation). Cluster 3 (sufficient ANC, else low) also showed a higher proportion of voucher recipients compared to nonrecipients by 5.5% points. On the contrary, the predicted probability of voucher recipients belonging to cluster 4 (overall low utilisation) was significantly lower (9.8%) compared to voucher non-recipients (41.9%) (Figure 2). The predicted probabilities of belonging to cluster 2 (high utilisation except ANC) are also lower among voucher recipients (5%) compared to voucher non-recipients (6.5%).

### Effect of voucher receipt on the probability of belonging to certain utilisation groups for the poor pregnant women

**Figure 3.**
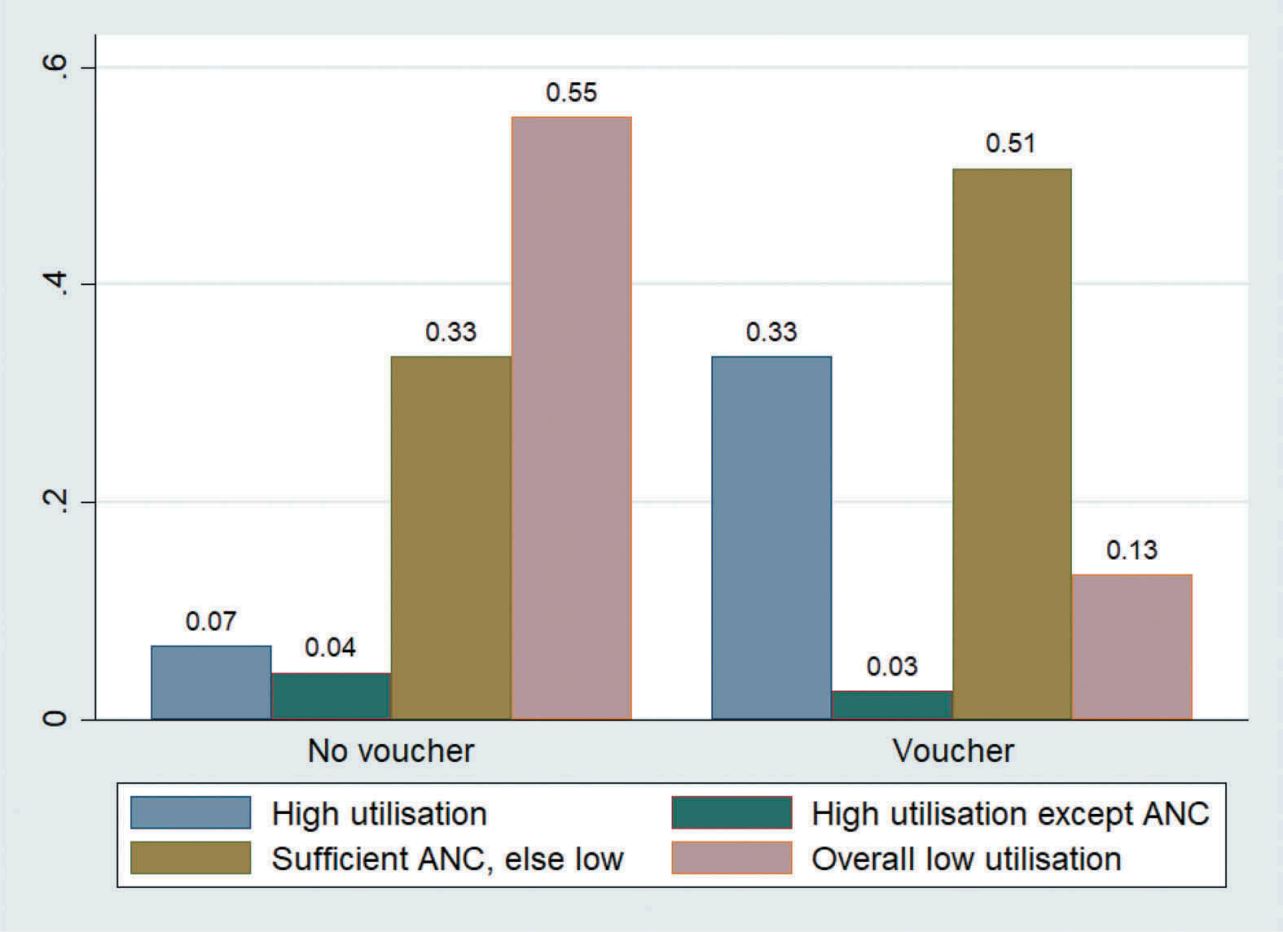
Predicted probabilities of cluster membership for bottom 2 asset quintiles.

The predicted probability analysis was re-run focusing only on women belonging to the bottom two asset quintiles, i.e. the poor pregnant women. Findings show that the predicted probability of non-voucher poor women belonging to the highest utilisation cluster was only 6.8% and the probability for the voucher recipients was higher at 33.3%. On the other hand, the probability of non-voucher poor women being in the lowest utilisation cluster was high at 55.4%, whereas the probability was only 13.3% for the poor women receiving vouchers (Figure 3).

## Discussion

Four different clusters of women were identified based on utilisation of all components of the CoC. Continuity in service utilisation sequentially decreased from one cluster to the next. 22.9% of the women belonged to the highest utilisation cluster who maintained the CoC and one-third of the women (33.3%) belonged to the lowest utilisation cluster with intermittent or discontinued use of services. Voucher (i.e. MHVS) receipt was found to enhance utilisation of the complete CoC and to significantly reduce the probability of being in a low-utilisation cluster. The effect of voucher receipt becomes more striking for poor pregnant women whose outcomes are of particular interest in the context of the current voucher scheme in Bangladesh. For them, the probability of voucher recipients belonging to the highest utilisation cluster was substantially higher relative to that of the non-voucher recipients (33.3% vs. 6.8%). By contrast, the probability of voucher-recipient poor women being in the low-utilisation cluster was much lower than the non-voucher poor women (13.3% vs. 55.4%). This, in turn, points to the financial barrier that women face in accessing maternal care which has been demonstrated in several earlier studies [13,25]. This is a key insight from our analysis, showing that the financial barrier is indeed key to non-utilisation and that demand-side financing schemes like vouchers can help pregnant women cross this significant barrier and attain access to the CoC. The favourable impact of vouchers on utilisation of each component of maternal care has been demonstrated in earlier studies carried out in Uganda, Kenya and Pakistan [14,26,27]. A systematic review of studies carried out in low- and middle-income countries confirmed the scope for voucher schemes to increase access to maternal care [28]. Studies carried out during the initial phase of the MHVS in Bangladesh found positive impacts on the utilisation of ANC, SBA at delivery, and PNC [21]. The current study enriched the global findings with its ability to explore the holistic effect of vouchers on the CoC. The analysis identified dropouts in the continuum who are essentially those forming clusters 3 and 4. Identifying the lack of linkage between the different components of care can assist countries struggling with low maternal health outcomes to design effective interventions that ensure continuity of maternal care replacing intermittent or partial care-seeking behaviour.

It was found that, alongside persistent gaps in coverage of individual components (with many women not receiving currently mandated levels of service for antenatal and delivery care) there is a substantial group of women with partial use of services across the CoC. Around 40% of women tends to use some ANC (1–2 ANC visits), but refrain from having facility delivery or delivery with an SBA and PNC. This is reflective of the current maternal care-seeking behaviour in rural Bangladesh where home delivery by traditional birth attendants with little or no formal training still remain the first preference [29]. The latest Bangladesh Demographic and Health Survey showed that 37% of the deliveries are assisted by untrained birth attendants [3]. This finding has useful implications for clinicians in terms of ensuring the provision of complete care for their clients. Interventions are needed that prompt clinicians to engage with women, families, and communities to discuss options for pregnancy and delivery care and emphasise the importance of all elements of care. Future research needs to be directed towards understanding the health-seeking behavior of service recipients in terms of their preference for the different services and the reasons behind these preferences. An integrated supply and demand-side approach has the potential to minimise the uncertainties and reservations around taking up institutional care arising from fear of medical intervention, an increasingly important barrier that has been reported in earlier studies carried out in rural Bangladesh and other countries with similar cultural context [21,30]. Further, it was found that even with vouchers, 43.5% women avail all components of care which warrants the need to explore the gaps in voucher implementation and uptake and thereby design interventions to ensure universal coverage of CoC.

Programmatic investments in Bangladesh have been historically directed towards financing individual service components such as SBA at delivery, child vaccination, and family planning. In recent years it has been realised that access to quality health services in pregnancy, childbirth and the postnatal period may yield multiple returns on investment by reducing maternal and neonatal deaths as well as improving child development outcomes [31,32]. Thus, maximum utilisation of all the components of maternal care can be anticipated to expedite progress towards attaining the Sustainable Development Goals for Bangladesh [33]. An emphasis on the CoC is exemplified by the Countdown approach of graphically presenting coverage data across the continuum [34]. Further, the newly launched National Social Security Strategy of Bangladesh includes scale-up of MHVS to extend proper social protection to the poor and vulnerable. At this stage of national planning, a coherent continuum-based approach to understanding maternal care-seeking behaviour, like the one presented in the current paper, can be anticipated to achieve a substantial impact.

Global policy and programme recommendations for maternal and newborn health continue to evolve: notably, the World Health Organization now advocates an eight-contact model for antenatal care [35]. In a setting where achieving full access in a much simpler three visit model and linkage to other elements of the care continuum has been incomplete, particular design and implementation strategies will be needed should Bangladesh move to take up the eight-contact model. A further noteworthy World Health Organization initiative has been to re-frame the concept of the ‘skilled birth attendant’ (as used in the present study) to ‘skilled health personnel’, drawing attention to the importance of professional care at each stage and more explicitly emphasizing the need for dedicated health staff such as midwives in these roles [36]. Operationalisation of this concept and impact assessment will require the type of analytical approach that has been used in this study.

One of the limitations of the current analysis is that it uses a proxy measure to assess the socio-economic status of the voucher recipients. The voucher programme in reality uses income and land ownership criterion for assessing the eligibility of women to be included in the scheme. In the absence of income data, the asset index was used instead. It is recognised that the group identified as poor and eligible for receipt of vouchers might differ to some extent with that of the voucher scheme. Further, the quality aspect of service delivery was not included extensively in our analysis. The number of visits for each component of care was considered, not the detailed elements of care offered at each of those visits.

## Conclusion

With the launch of a national midwifery programme in Bangladesh, there is an opportunity to take a continuum-based approach to scale. The particular emphasis in professional midwifery practice is on the CoC and this has real potential to address the gaps that are identified through our analysis. The fact that a country that has experienced a miracle in terms of fertility reduction over a period of four decades is still facing challenges to bring the majority of its women to the health facilities for safer delivery remains a mystery. A holistic approach is expected to explain the gaps and identify possible solutions towards a maternal health transition and better birthing experience for women in Bangladesh and in other countries with similar context.

## Data Availability

The authors confirm that all data underlying the findings in this paper are freely available upon request subject to the adherence to icddr,b’s data sharing policy (http://www.icddrb.org/policies). The request for the data can be sent to shaila@icddrb.org, the Principal Investigator of the project.

## Paper context

Women in developing countries lack access to comprehensive maternal care, a risk factor for high maternal mortality. Continuum-of-care is known as a key programme strategy for reducing maternal deaths. The cluster analysis used in the current paper showed that vouchers encouraged participation in the CoC, particularly for poor women. The analysis identified drop outs in the CoC. Successful interventions to ensure CoC should rely on a better understanding of the gaps in care-seeking along CoC.
